# Evaluating a Novel Cell‐Free Preservation Solution for Human Cardiomyocyte Protection: A Proof‐of‐Concept Study

**DOI:** 10.1155/bmri/7101969

**Published:** 2026-07-04

**Authors:** Christian Beckers, Sandra Kraemer, Alexander Theißen, Merlin Kunze, Josefin Soppert, Leon Schurgers, Rogier Veltrop, Benedict Doorschodt, René H. Tolba, Jan Larmann, Christian Bleilevens

**Affiliations:** ^1^ Department of Anaesthesiology, Medical Faculty, University Hospital RWTH Aachen, Aachen, Germany, ukaachen.de; ^2^ Department of Intensive Care, Medical Faculty, University Hospital RWTH Aachen University, Aachen, Germany; ^3^ Institute for Laboratory Animal Science and Experimental Surgery, Medical Faculty, RWTH Aachen University, Aachen, Germany, rwth-aachen.de; ^4^ Department of Heart Surgery, Medical Faculty, University Hospital RWTH Aachen, Aachen, Germany, ukaachen.de; ^5^ Department of Biochemistry, Cardiovascular Research Institute Maastricht, Maastricht University, Maastricht, Netherlands, maastrichtuniversity.nl; ^6^ Vivalyx GmbH, Cologne, Germany

**Keywords:** cardiomyocytes, cell free, normothermic, organ preservation

## Abstract

**Background:**

Heart transplantation remains the definitive therapy for end‐stage heart failure; however, its clinical impact is constrained by donor availability and preservation‐associated injury. Static cold storage (SCS) with cardioplegic solutions such as Custodiol is the current clinical standard of care, yet extended cold ischemia markedly increases the risk of primary graft dysfunction. There is therefore a critical need for advanced preservation strategies and solutions that maintain graft viability under both cold and normothermic conditions.

**Aim:**

This study is aimed at comparing the effects of Custodiol and the novel cell‐free organ preservation solution Omnisol on contractile function, metabolic activity, and apoptosis in human cardiomyocytes under static cold and normothermic storage conditions.

**Materials and Methods:**

Human‐induced pluripotent stem cell–derived cardiomyocytes were subjected to static cold or normothermic storage in standard culture medium, Custodiol, or Omnisol, followed by functional assessment after return to standard culture medium. Contractile function was quantified using video‐based motion analysis (MuscleMotion), metabolic activity was quantified by MTT assay, and cell death–associated DNA fragmentation was assessed by TUNEL staining in combination with cell morphology.

**Results:**

Following normothermic preservation in Omnisol, cardiomyocyte contractility was comparable to that observed with standard culture medium, whereas Custodiol‐preserved cells showed markedly reduced beating frequency, impaired contraction profiles, and delayed functional recovery. After SCS, contractile function recovered to a similar extent in Omnisol‐ and Custodiol‐treated cells upon return to standard medium. Importantly, Omnisol‐treated cardiomyocytes displayed significantly higher metabolic activity than Custodiol‐treated cells under both cold and normothermic storage conditions. Apoptosis rates were comparable between groups following cold storage; however, under normothermic conditions, Custodiol treatment was associated with a significant increase in apoptotic cell death.

**Conclusion:**

This proof‐of‐concept study demonstrates the preservation potential of the novel cell‐free preservation solution Omnisol for human cardiomyocytes under static cold and normothermic conditions. These hypothesis‐generating findings support further evaluation of Omnisol as a versatile preservation solution for dynamic organ perfusion strategies.

## 1. Introduction

Heart transplantation remains the definitive treatment for end‐stage heart failure; however, its application is constrained by the limited availability of suitable donor organs [1]. A critical component of the transplantation process is the preservation of the donor heart from procurement to implantation. For over 6 decades, static cold storage (SCS) has been the established gold standard, involving organ flushing with a cold cardioplegic solution and subsequent transportation on ice [2]. This method reduces cellular metabolism but inevitably leads to time‐dependent ischemic and cold‐induced injuries. Prolonged cold ischemic time (CIT), particularly beyond 3–4 h, is significantly associated with an increased risk of primary graft dysfunction (PGD), which is the leading cause of early posttransplant morbidity and mortality [1, 3]. Despite these risks, a substantial proportion of hearts are transplanted with a CIT exceeding 4 h, underscoring the need for improved preservation technologies [4].

In response to the limitations of SCS, a paradigm shift towards dynamic preservation modalities is ongoing, with the aim of mitigating ischemic injury, extending preservation times, and expanding the donor organ pool [5]. This shift has introduced several novel technologies into clinical practice. One approach is controlled hypothermic storage, as enabled by the Paragonix SherpaPak system, which maintains the donor heart at a consistent temperature range of 4°C–8°C, thereby avoiding excessive cooling and potential freeze‐induced injury associated with traditional ice storage [6].

Another major advancement is machine perfusion (MP), which provides continuous perfusion to the donor heart during transport. MP technologies are broadly categorized into two categories. Normothermic machine perfusion (NMP) is exemplified by the Transmedics Organ Care System (OCS). It maintains the organ in a near‐physiologic heart‐beating state using warm, oxygenated, and nutrient‐enriched donor blood. This allows for continuous metabolic support and functional assessment of the graft ex vivo, which has been crucial in enabling the successful transplantation of hearts from donors after circulatory death (DCD) and extended criteria donors (ECD), as published during the PROCEED II trial in 2015 [7]. The alternative, hypothermic oxygenated perfusion (HOPE), also known as nonischemic heart preservation (NIHP), combines the metabolic suppression of hypothermia with continuous oxygenated perfusion to minimize ischemic injury. Preclinical and clinical studies suggest that HOPE can significantly reduce the incidence of PGD [8]. These innovative preservation strategies are transforming heart transplantation by potentially improving patient outcomes, increasing the utilization of donor organs, and allowing safer transplantation of high‐risk and geographically distant hearts.

While Heart‐NMP is performed with donor blood as perfusate [9], within the NIHP 2019 study, a special heart solution (XVIVO Heart Solution) containing red blood cells was used as perfusate [10]. Custodiol solution is the gold standard for SCS in multiorgan compatibility but is predominantly used for hearts and as a cardioplegic solution [11].

The aim of this study was to compare Custodiol with a new cell‐free organ preservation solution, Omnisol, for storing human‐induced cardiomyocyte (iCM) cell cultures. Omnisol was developed with the focus on abdominal organ preservation of kidney, liver, and pancreas. The composition of Omnisol is optimized for SCS and HOPE, as well as NMP of abdominal organs, and due to its composition, it is potentially beneficial for cardiomyocyte preservation. In general, cell‐free organ preservation solutions minimize blood‐related complications, like immune reactions in the recipient and subsequent graft rejection.

## 2. Materials and Methods

### 2.1. Induced Pluripotent Stem Cell (iPSCs) Culture

Human peripheral blood mononuclear cell (PBMC) derived iPSCs from a healthy donor (CARIM ‐ i007B female) were cultured in mTESR Plus medium (Stemcell, 100‐0276) on Matrigel‐coated (Corning, 354277) six‐well plates and passaged with 0.5 mM EDTA dissociation buffer (0.5 mmol/EDTA, Sigma E5134) in DPBS (Gibco 14190144) for a minimum of four passages prior to differentiation. At 75%–80% confluence, iPSCs were differentiated into iCMs using a small‐molecule protocol [12], consisting of incubation in RPMI media including 2% insulin‐free B27 (Thermo Fisher Scientific, A1895601) and 8 *μ*M CHIR99021 (Selleckchem, S1263) for 48 h, followed by incubation with RPMI with 2% insulin‐free B27 and 2 *μ*M WNT C‐59 (Toris, 5148) for 48 h. The iPSCs were then cultured in RPMI (Thermo Fisher Scientific, 11875) with 2% B27 (Thermo Fisher Scientific, 17504044) with media changes every other day for 10–12 days. On Days 10–12, metabolic selection was induced by cultivation in glucose‐ and pyruvate‐free DMEM (Thermo Fisher Scientific, A1443001) with 2% B27 and 5 mM L‐lactate (Sigma, 71718) for 3–4 days. Subsequently, iCMs were dissociated with 10x TrypLE (Thermo Fisher Scientific, A1217701) for 15–45 min and replated on 12‐well plates coated with Matrigel (Corning, 354277) and cultivated in glucose‐free RPMI with 2% B27, 10% KnockOut Serum Replacement (Thermo Fisher Scientific,10828) and 1% RevitaCell Supplement (Thermo Fisher Scientific, A2644501) for 24 h. For TUNEL staining, cells were plated on Matrigel‐coated *μ*‐Dishes 35 mm (Ibidi, 81156). Metabolic selection was repeated for 3–4 days. The iCMs were matured for 10–14 days by culturing in metabolic maturation medium, with the medium changed every other day (Table S1). It should be noted that metabolic characterization of the iCMs following the MMM protocol was not formally assessed in the present study. Based on current knowledge of iPSC‐CM biology [13, 14], these cells likely retain a predominantly glycolytic, neonatal‐like metabolic phenotype with inherently greater ischemia tolerance than adult ventricular cardiomyocytes.

### 2.2. Incubation Protocol With Custodiol Versus Omnisol

**Figure 1:**
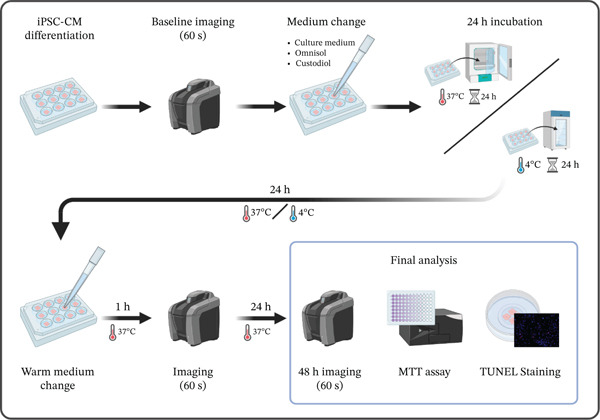
Experimental workflow for evaluating preservation solutions in human iPSC‐derived cardiomyocytes. After iPSC‐CM differentiation, baseline imaging was performed, followed by medium exchange to culture medium, Omnisol, or Custodiol. Cells were then incubated for 24 h at either 37°C or 4°C. After 24 h, all groups underwent warm medium change and were reincubated at 37°C for 24 h, with imaging performed after 1 h and at 48 h. Final analysis included live imaging, MTT assay, and TUNEL staining.

To investigate the preservation potential of Custodiol or Omnisol, the cardiomyocyte medium (Gibco), which represents physiological conditions, was replaced under two different storage conditions for both preservation solutions: for 24 h at 37°C to mimic normothermic conditions or 24 h at 4°C to mimic cold storage conditions. Subsequently to the preservation phase, solutions were replaced with the original cell culture media, and the first measurement was performed after 1 h, followed by another 24 h incubation period in standard medium, for a second window of analysis after 48 h. The exact incubation protocol, including video capture slots to measure the functionality of the cardiomyocytes, as well as the 12‐well format chosen for the different analytical methods, is shown in Figure 1. To translate this protocol to a clinical situation, the cell culture medium represents the donor, whereas the preservation solutions mimic the ex vivo storage conditions, and the subsequent change back to the cell culture medium represents reperfusion after transplantation.

### 2.3. Video‐Based Contractility Measurements

Videos of the iCM cell cultures were captured using a Keyence microscope (Keyence BZ‐X800, Frankfurt, Germany), in combination with a compatible cell culture incubation chamber (OKOLAB Uno‐T‐H‐CO2, Naples, Italy). Video sequences of 60 s, at 29 frames per second with a temporal resolution of 34 ms per frame, were taken at baseline (BL), after 24 or 48 h of warm or cold storage, followed by a back‐change to warm standard culture medium for 1 h prior to the video capturing. Video analysis was performed using a published script for ImageJ software [15]. The analyzed parameters were contraction in beats per minute (BPM), contraction duration, time to peak, and relaxation time (ms) according to a previously published protocol [15].

### 2.4. MTT Assay

The metabolic activity was measured using an MTT assay. Briefly, 200 *μ*L of MTT Solution (5 mg/mL Methylthiazolyldiphenyl‐tetrazoliumbromid [Sigma, M2128] in PBS) was added per 1 mL of medium in each well and incubated at 37°C for 10–15 min. A lysis control with 0.1% Triton X100 (Carl Roth, 3051.3) in PBS (Thermo Fisher Scientific, 14190144) was prepared. After incubation, the medium was removed, and the cells were lysed with 1.5 mL DMSO (Sigma, D8418) per well for 10 min. A total of 200 *μ*L was transferred to a 96‐well plate, and the optical density (OD) values were measured at 550 nm using a plate reader (Tecan, Infinite 200 Pro). Cell viability was calculated using the following formula:

%MTT reduction=OD sample−OD lysisOD medium−OD lysis ×100.

The MTT assay measures mitochondrial reductase activity as a proxy for metabolic activity and does not directly distinguish between increased metabolic rate per cell and changes in cell number, nor does it provide information on respiratory coupling or ATP/ADP ratios.

### 2.5. TUNEL Assay

Briefly, the incubation medium was removed from each dish and stored for further analysis, and the dishes were washed twice with 1 mL PBS. Cells were fixed with 4% PFA (Sigma, HT501128) for 1 h, then washed twice with PBS. After permeabilization with 0.1% Triton X‐100 in PBS for 2 min, cells were washed twice with PBS. Cells were stained with 100 *μ*L of TUNEL reaction mix (In Situ Cell Death Detection Kit TMR red, Roche, 12156792910) for 1 h at 37°C. The cells were washed twice with PBS, mounted with DAPI Fluoromount‐G (SouthernBiotech, 0100‐20), and stored at 4°C overnight or until imaged.

Imaging was performed using a Keyence BZ‐X800 microscope and automated optimal settings for the blue (DAPI) and red (TUNEL) channels. Analysis was performed using the Hybrid Cell Count tool in the BZ‐X800 Analyzer software. From one image, parameters were defined and saved as macros, and all images were analyzed.

### 2.6. Statistics

Statistical analysis and graph design were performed using GraphPad Prism Software (Version 9.2.0, GraphPad Software, Inc., La Jolla, California, United States). As the data were not normally distributed, a nonparametric Kruskal–Wallis test including Dunn′s multiple comparison was performed. Data are presented as boxplots, with the median value and min to max whiskers. Differences were considered significant at *p* < 0.05.

## 3. Results

### 3.1. Comparable Cardiomyocyte Contractility Under Normothermic Storage With Omnisol and Standard Culture Medium

**Figure 2:**
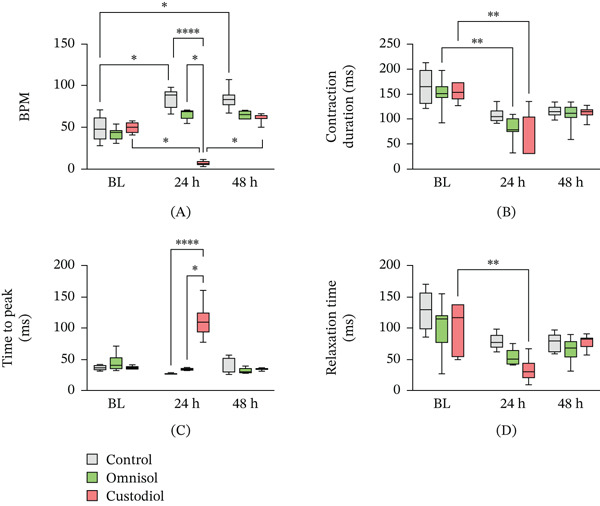
Contraction of iCMs under normothermic storage conditions. (A) Assessment of beating frequency in beats per minute (BPM), (B) contraction duration in milliseconds (ms), (C) time to peak of contraction, and (D) relaxation time until the next contraction in the control group treated for 48 h under static cold storage conditions with standard culture media (gray), Omnisol (green), or the clinical gold standard Custodiol (red).  ^∗^ *p* < 0.05;  ^∗∗^ *p* < 0.01. *n* = 2 biological replicates, with each quadruple technical replicates.

After 24 h of normothermic storage, iCMs cultured in standard culture medium (control) exhibited a significant increase in beating frequency compared to BL (49 ± 15 vs. 85 ± 11 bpm, *p* = 0.023). This increase was accompanied by a significant reduction in contraction duration (165 ± 35 vs. 107 ± 15 ms, *p* = 0.003) and a tendency towards reduction of the relaxation time (128 ± 31 vs. 79 ± 12 ms, *p* = 0.7). This phenotype of accelerated but smaller‐amplitude contractions persisted after 48 h (Figure 2A–D).

Similarly, iCMs kept in Omnisol displayed a slight, nonsignificant increase in beating frequency during 24 h of normothermic storage, along with reduced contraction duration and relaxation time. These parameters remained largely unchanged after transfer back to standard culture medium and incubation at 37°C for another 24 h (Figure 2A–D). Contractile parameters were comparable to those of the control group, indicating noninferiority of Omnisol relative to standard culture medium under normothermic conditions.

iCMs preserved in Custodiol showed a significant decrease in beating frequency compared with BL (49 ± 7 vs. 7 ± 3 bpm, *p* = 0.05) under normothermic storage conditions after switchback to standard culture medium (Figure 2A). Custodiol‐cultured iCMs exhibited a markedly dampened contraction profile, with only a limited number of detectable contractions within the recording frames, precluding reliable quantitative analysis of contractile parameters (Figure 2B–D). Interestingly, the contraction recovered after an additional 24 h incubation in culture medium. As Custodiol is not formulated for normothermic use, this impairment represents the expected consequence of its composition, and the normothermic Custodiol condition is appropriately interpreted as a negative control rather than a comparative benchmark for Omnisol efficacy.

### 3.2. Comparable Cardiomyocyte Contractility Under SCS With Omnisol and Custodiol

**Figure 3:**
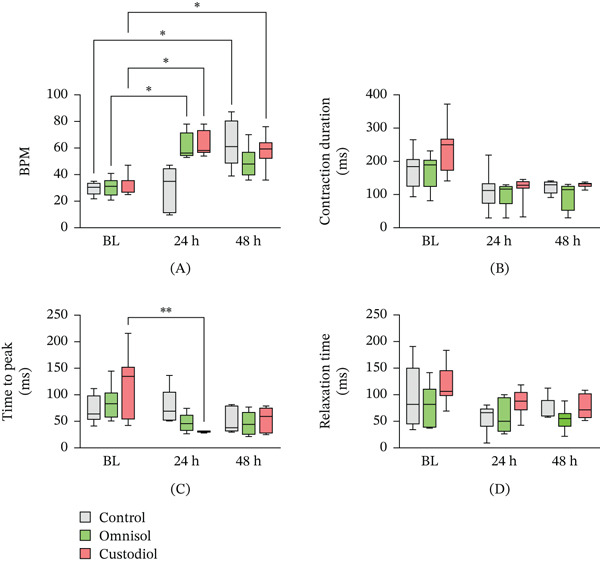
Contraction of iCMs under static cold storage conditions. (A) Assessment of beating frequency in beats per minute (BPM), (B) contraction duration in milliseconds (ms), (C) time to peak of contraction, and (D) relaxation time until the next contraction in the control group treated for 48 h under static cold storage conditions with standard culture media (gray), Omnisol (green), or the clinical golden standard Custodiol (red).  ^∗^ *p* < 0.05;  ^∗∗^ *p* < 0.01. *n* = 2 biological replicates, with each quadruple technical replicates.

After 24 h of SCS, only control iCMs maintained in standard culture medium exhibited contractile parameters comparable with BL (Figure 3A). In contrast, iCMs preserved in Omnisol (31 ± 7 vs. 61 ± 10 bpm, *p* = 0.029) or Custodiol (31 ± 8 vs. 63 ± 10 bpm, *p* = 0.012) showed a significantly increased beating frequency accompanied by a trend towards reduced contraction duration and shortened time to peak. After 48 h, contractile parameters appeared to recover and were comparable across all culture conditions (Figure 3B–D).

### 3.3. Comparable Preservation of Cardiomyocyte Metabolic Activity by Omnisol and Standard Culture Medium Under Warm and Cold Conditions

**Figure 4:**
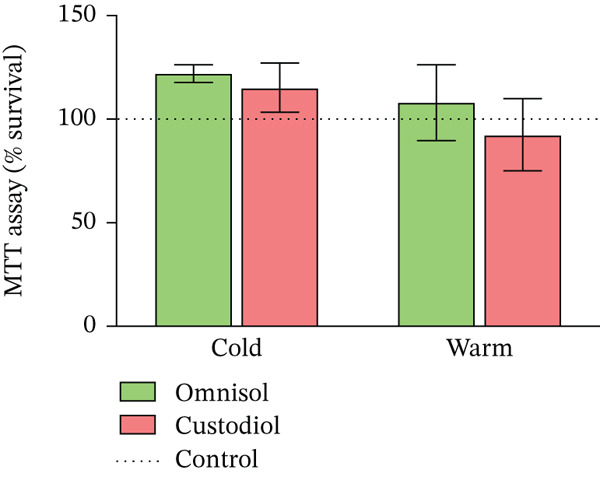
Survival of the iCMs during MTT Test in the control group (100% dashed line) and Omnisol‐treated cells (green) for normothermic (*n* = 5) and static cold‐stored cells (*n* = 5) in comparison to Custodiol‐treated cells (red) after 48 h. *n* = 2 biological replicates, with one technical triplicate and one technical duplicate (*n* = 5 per group in total).

Although contractile function appeared to recover after an additional 24 h of culture at 37°C, independent of storage temperature (cold vs. normothermic) and preservation solution, we next assessed whether metabolic activity and apoptosis differed between conditions. Metabolic activity tended to be higher in Omnisol‐treated iCMs under both SCS and normothermic conditions compared with Custodiol (normothermic: 108*%* ± 40*%* vs. 93*%* ± 39*%*, cold: 122*%* ± 9*%* vs. 115*%* ± 26*%*) (Figure 4).

**Figure 5:**
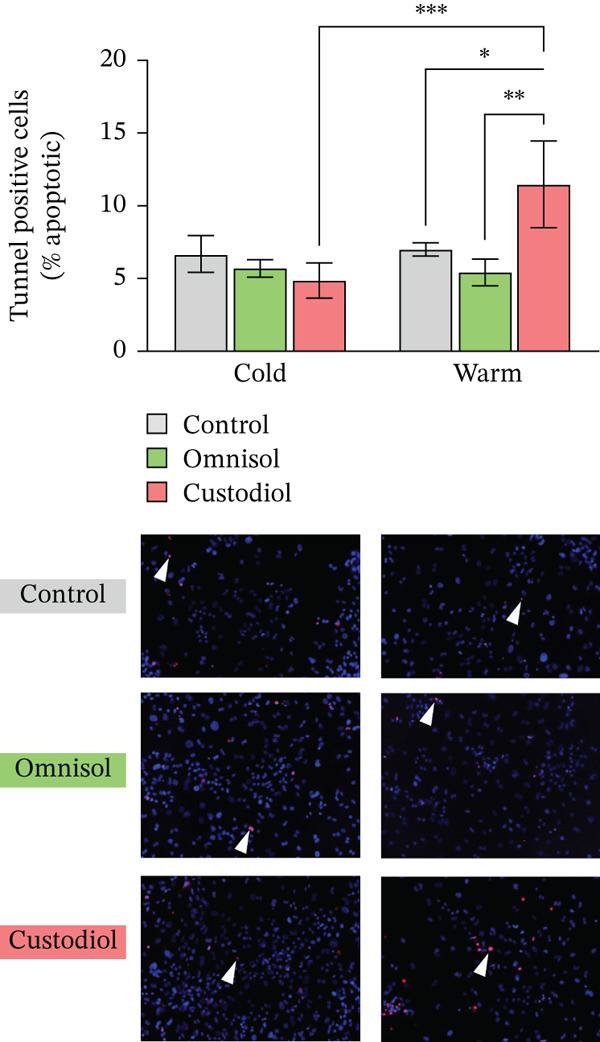
Tunnel‐positive stained iCMs (white arrow) indicate apoptotic cells for normothermic (warm) and static cold stored (cold) conditions in control (gray), Omnisol (green), or Custodiol (red) treated cells after 48 h  ^∗^ *p* < 0.05;  ^∗∗^ *p* < 0.01;  ^∗∗∗^ *p* < 0.001. *n* = 1 biological replicate, with five technical replicates per group (*n* = 5 per group in total).

The percentage of apoptotic iCMs was comparable across all preservation solutions under SCS, whereas apoptosis was significantly increased in Custodiol‐treated iCMs under normothermic conditions (Custodiol: 12*%* ± 3*%* vs. Omnisol: 5*%* ± 2*%*; *p* = 0.003 vs. control: 7*%* ± 0.5*%*, *p* = 0.049) (Figure 5). These normothermic apoptosis data are derived from one biological replicate with five technical replicates per group and should therefore be interpreted with appropriate caution. In summary, Omnisol tends to improve metabolic activity and limit apoptosis, particularly under normothermic storage conditions.

## 4. Discussion

The present proof‐of‐concept study demonstrates that human iPSC‐derived cardiomyocytes can be maintained in cell culture under both warm and cold conditions when standard culture medium is replaced for 24 h with either Omnisol or Custodiol. The key findings can be summarised as follows. Under normothermic conditions (37°C), iCMs preserved in Omnisol maintained contractile parameters that were noninferior to standard culture medium controls, whereas Custodiol‐treated cells (Custodiol is explicitly intended for cold storage only) showed markedly impaired contractility at 24 h with recovery at 48 h. Under SCS, both Omnisol and Custodiol resulted in a comparable rebound in beating frequency at 24 h, with convergence to control levels at 48 h. Both preservation solutions therefore performed equivalently under cold conditions. After 48 h under normothermic conditions, Custodiol‐treated cells showed a significantly increased proportion of TUNEL‐positive cells, consistent with the known incompatibility of this formulation with warm storage, whereas Omnisol‐treated cells were comparable to the control group under both temperature conditions.

For more than 6 decades, SCS using cardioplegic solutions, such as Custodiol, remained the clinical gold standard for heart preservation before transplantation [16, 17]. Although SCS is simple and robust, its intrinsic reliance on hypothermic ischemia imposes strict temporal limits and does not fully prevent anaerobic metabolism, accumulation of toxic metabolites, and subsequent ischemia–reperfusion injury [2, 8]. These shortcomings become particularly evident when the CIT exceeds 4 h, a recently described threshold determining heart and lung transplants as high‐risk organs beyond this ischemia time, with an increased risk of PGD [18].

Against this background, the development of novel cell‐free organ preservation solutions capable of supporting both SCS and dynamic MP represents a paradigm shift in cardiac transplantation [6] [2]. In contrast to traditional intracellular‐type cardioplegic solutions, modern extracellular, hyperoncotic, and nutrient‐enriched formulations, such as the XVIVO Heart Solution, are designed to maintain endothelial integrity, buffer metabolic derangements, and allow controlled oxygen delivery across a wide temperature range. Importantly, such solutions are not limited to cold cardioplegia but can function as true perfusates, bridging hypothermic preservation and more metabolically active preservation strategies, such as NMP [7] or HOPE [19].

Under SCS, both Omnisol and Custodiol resulted in an increased beating frequency at 24 h compared with BL, whereas the control group maintained stable beating frequency at 24 h and only reached similarly elevated levels at 48 h.

Notably, both preservation solutions may provide a more stable cellular environment during cold storage compared with standard culture medium, possibly related to their improved buffering capacity, osmotic stabilization, and potential attenuation of reactive oxygen species (ROS) accumulation [20–23]. Standard cell culture medium lacks several components present in dedicated organ preservation solutions, including specific amino acids, ROS scavengers, colloid‐oncotic agents, and enhanced buffer capacity. It is important to note, however, that the underlying mechanisms, such as oxidative stress modulation, mitochondrial preservation, and ion channel stabilization, were not directly assessed in the present study. The observed effects may reflect improved metabolic and oxidative stability during cold storage, but this interpretation requires direct experimental validation in future studies.

Under normothermic conditions, iCMs contraction performance was well preserved in the standard culture medium, as well as in Omnisol, whereas Custodiol‐preserved iCMs showed reduced function at 24 h and delayed recovery after 48 h.

As explicitly stated in the product specification, Custodiol is not formulated for normothermic preservation. Its inferior performance under warm conditions is therefore an expected consequence of its composition, specifically the high potassium concentration and the absence of metabolic substrates for aerobic energy production, rather than evidence of Omnisol superiority per se. The relevant normothermic comparison is Omnisol versus standard culture medium, and the present data support noninferiority of Omnisol in this context.

The potential benefit of Omnisol for cardiomyocyte preservation, especially in the warm condition, may derive from its more physiologically balanced, cell‐free composition rather than from a single cardioprotective component. Compared with Custodiol, containing very low sodium and calcium together with high histidine buffering capacity and potassium concentration, Omnisol provides higher amounts of calcium, balanced with adapted amounts of potassium and sodium, in combination with antioxidants like taurine (Table S2). This potentially provides an optimized constitution for cardiomyocyte preservation, hypothetically by maintaining osmotic and ionic stability, limiting depolarization‐associated calcium dysregulation, supporting metabolic activity. Especially calcium overload is a central mechanism of myocardial ischemia–reperfusion injury, and disturbances in intracellular calcium homeostasis directly affect both contraction and electrical stability [24], as referred to as pharmacological approaches to finetune the mitochondrial calcium handling by Bertero et al. [25].

In assessing apoptosis and metabolic activity, we could not demonstrate a general proapoptotic effect under SCS conditions in any of the groups, consistent with the known cytoprotective role of hypothermia [26]. Regarding normothermic conditions, significantly increased numbers of TUNEL‐positive cells were detected in the Custodiol group only, consistent with the cellular toxicity of this cold‐only formulation at 37°C. The increased apoptosis in the Custodiol normothermic group did not translate into significantly reduced MTT values, a discrepancy that likely reflects the temporal offset between early apoptotic signaling (detectable by TUNEL) and the broader metabolic changes captured by MTT.

As this study isolates cardiomyocyte‐specific responses in a monolayer cell culture model and does not capture vascular, endothelial, immune, or perfusion‐dependent mechanisms that are relevant to whole‐organ preservation, the present findings are therefore best interpreted as hypothesis generating, providing a cellular rationale for further investigation of Omnisol in more complex experimental systems, such as engineered cardiac tissue constructs [23] or isolated ex vivo heart models [27].

Summarizing, our findings from the SCS setting, they are in a row with recently published data by Freitas‐Ribeiro et al. [26], who described that hypothermia, respectively, SCS protects the extracellular matrix. Thus, SCS in any of our test media seems to be a cell protective strategy, with comparable results towards standard cell culture medium, whereas Omnisol can be used for warm and cold cardiomyocytes storage.

Nevertheless, we have to mention several limitations of our study. First, it is a cell‐culture approach with low translational potential into a clinical scenario like a beating heart scenario. Second, the biological replicates are low (*n* = 2 per group and condition), and due to the proof‐of‐concept nature of this study, we were not able to perform additional experiments. However, with the technical quadruple per group and condition, we could show low internal deviation between the replicates, which leads to a sufficient power to achieve relevant results. Third, the intended use of Custodiol is SCS only and explicitly not normothermic preservation, which in turn means expectable inferior results in the normothermic group. However, this is a proof of concept for applying Omnisol in warm and cold storage settings for cardiomyocytes. Fourth, lacking analytical methods like calcium transient analysis and multielectrode array recordings, and oxidative capacity measurements only enable a hypothetical interpretation of potential preservation capabilities of the preservation solutions. Fifth, the iPSCs applied in this study are predominantly of a neonatal‐like phenotype with increased ischemia tolerance compared with mature cardiac myocytes.

## 5. Conclusion

This proof‐of‐concept study demonstrates that the novel cell‐free preservation solution Omnisol has the potential to maintain human cardiomyocyte function and viability under both static cold and normothermic storage conditions. Under normothermic storage, Omnisol‐treated cells were noninferior to standard culture medium controls in terms of contractility, apoptosis, and metabolic activity. The predictably impaired performance of Custodiol under normothermic conditions, consistent with its intended use for cold storage only, underscores the value of more versatile preservation solutions for dynamic perfusion strategies. These hypothesis‐generating findings support further evaluation of Omnisol in more physiologically experimental models.

## Author Contributions

Conceptualization: Christian Beckers and Christian Bleilevens. Methodology: Christian Beckers, Sandra Kraemer, Alexander Theißen, Merlin Kunze, and Josefin Soppert. Validation: Leon Schurgers and Rogier Veltrop. Formal analysis: Christian Beckers and Christian Bleilevens. Investigation: Christian Beckers, Alexander Theißen, and Merlin Kunze. Resources: Benedict Doorschodt, René H. Tolba, and Jan Larmann. Data curation: Christian Beckers. Writing—original draft preparation: Christian Beckers and Christian Bleilevens. Writing—review and editing: Benedict Doorschodt, Sandra Kraemer, Alexander Theißen, Merlin Kunze, Josefin Soppert, Leon Schurgers, Rogier Veltrop, René H. Tolba, Jan Larmann, and Christian Bleilevens. Visualization: Christian Beckers and Christian Bleilevens. Supervision: Benedict Doorschodt, René H. Tolba, and Christian Bleilevens. Project administration: Christian Bleilevens.

## Funding

No funding was received for this manuscript.

## Disclosure

All authors have read and agreed to the published version of the manuscript.

## Conflicts of Interest

Benedict Doorschodt is the founder and chief technology officer of Vivalyx GmbH. Christian Bleilevens is the founder and chief scientific officer of Vivalyx GmbH. René H. Tolba is the founder and chief medical officer of Vivalyx GmbH. The other authors declare no conflicts of interest.

## Supporting information


**Supporting Information** Additional supporting information can be found online in the Supporting Information section. Table S1 provides the exact composition of the metabolic maturation medium, described briefly in the Section 2. Table S2 provides the composition of Omnisol and Custodiol preservation solutions.

## Data Availability

The data that support the findings of this study are available from the corresponding author upon reasonable request.
