# Supplementary material for: Evaluating a Novel Cell‐Free Preservation Solution for Human Cardiomyocyte Protection: A Proof‐of‐Concept Study
Source: Biomed Res Int. 2026 Jul 4;2026:7101969. doi: 10.1155/bmri/7101969 (PMC13332395; doi:10.1155/bmri/7101969)
Supplement: Supplementary file 1 — Supporting Information Additional supporting information can be found online in the Supporting Information section. Table S1 provides the exact composition of the metabolic maturation medium, described briefly in the Section 2. Table S2 provides the composition of Omnisol and Custodiol preservation solutions. [file BMRI-2026-7101969-s001.pdf]

Supplemental:

Table 1: Composition of the Metabolic Maturation Medium, applied to the iCMs for 10-14 days of maturation time.

**Metabolic Maturation Medium**	**Order No.**	**mg/500 ml**
DMEM w/o Glucose	Thermo Fisher, 11966025	500 ml
3 mM Glucose	Merck, 1.08342.2500	270.2 mg
10 mM L-Lactate	Sigma, 71718	560 mg
5 µg/ml Vitamin B12	Sigma, V6629	50 µl
0.82 mM Biotin	Sigma, B4639	100.2 mg
5 mM Creatine monohydrate	Sigma, C3630	37.3 mg
2 mM Taurine	Sigma, T0625	
2 mM L-Carnitine	Sigma, C0283	197.7 mg
0.5 mM L-ascorbic acid	Sigma, A8960	72.4 mg
1x NEAA	Thermo Fisher, 11140050	5 ml
1x Linoleic Acid-Oleic Acid-Albumin	Sigma, L9655	5 ml
1x B27 Supplement	Thermo Fisher, 17504044	10 ml
1 % KnockOut Serum Replacement	Thermo Fisher,10828	5 ml

Table 2: Composition of Omnisol and Custodiol preservation solution

**Component**	**Ingredients**	**Omnisol (g/l)**	**Custodiol (HTK) (g/l)**
Colloid	PEG 35,000	18.000	
Buffers	Magnesium gluconate	2.880	
	Calcium gluconate	0.250	
	Calcium Chloride Dihydrate		0.002
	Lactobionic acid	3.940	
	HEPES	6.000	
	Potassium Hydrogen2-oxoglutarate		0.180
	Potassium Chloride	0.890	0.670
	Sodium Chloride	1.100	0.880
	Sodium gluconate	12.800	
Antioxidants	Taurine	2.000	
Energy substrates	Trehalose	0.500	
	Glucose	0.900	
	Mannitol		5.470
Amino acids	Tryptophan, Arginine, Histidine, Cysteine, Glutamic acid, Glutamine, Glycine	Yes (all)	Yes (Histidin, Tryptophan)
Viscosity	at approximately room temperature	~1.5-3.0 c.P.	~1.2 c.P.
Osmolarity		~320 mOsmol/L	~310 mOsmol/L
